# The influence of self-esteem and therapeutic alliance on psychotic symptom severity

**DOI:** 10.1192/j.eurpsy.2025.2272

**Published:** 2025-08-26

**Authors:** N. Middelkoop, M. Tetzlaff, S. Castelein, J. Bruins

**Affiliations:** 1 Research, Lenits Psychiatric Institute; 2 Department of mood disorders, PsyQ; 3 Faculty of Behavioral and Social Science, University of Groningen; 4 Rob Giel Research center, University Medical Center Groningen, University Center for Psychiatry, Groningen, Netherlands

## Abstract

**Introduction:**

The therapeutic alliance (TA) is increasingly acknowledged as a fundamental quality of care indicator. Numerous guidelines advocate TA awareness in practice, but lack specifics on building a strong TA. Yet, previous studies have found independent associations between levels of self-esteem, the quality of TA and severity of clinical symptoms in people with schizophrenia and other psychotic disorders. It suggests that the TA possibly mediates the relationship between self-esteem and psychotic symptoms. The present study therefore examined the relationships between these three factors in people with psychotic disorders.

**Objectives:**

Investigating the mediating effect of TA on the relationship between self-esteem and psychotic symptom severity.

**Methods:**

The short forms of the *Self-Esteem Rating Scale* and the *Working Alliance Inventory*, respectively, were used to assess self-esteem and TA. Psychotic symptoms were evaluated using the *Positive and Negative Syndrome Scale.* Linear regression models were applied, followed by a mediation-model when appropriate.

**Results:**

A higher self-esteem significantly predicted less severe psychotic symptoms (*B* = -.312; *β* = -.46, *p* <.001) and better TA (*B* = .123, *β* = .255, *p =* .009). There was no significant relation between TA and psychotic symptom severity (*B* = -.161; *β* = -0.109, *p* = .289), therefore no mediation-analysis was performed.

**Conclusions:**

We found no association between TA and psychotic symptoms, which may be explained by the mild psychotic symptoms and overall high satisfaction scores on TA in our chronic sample. Another factor might be that current measurements assume a one-on-one relationship between a client and a professional, while nowadays multiple professionals are involved. We recommend re-evaluating the definition and assessment of the TA within chronic psychiatric populations. Our study results also offer practical guidelines for clinicians to improve their quality of care, such as the recommendation to focus on enhancing self-esteem in people with psychosis.

**Disclosure of Interest:**

None Declared

